# Silver Nanoparticles Surface-Modified with Carbosilane Dendrons as Carriers of Anticancer siRNA

**DOI:** 10.3390/ijms21134647

**Published:** 2020-06-30

**Authors:** Elżbieta Pędziwiatr-Werbicka, Michał Gorzkiewicz, Katarzyna Horodecka, Viktar Abashkin, Barbara Klajnert-Maculewicz, Cornelia E. Peña-González, Javier Sánchez-Nieves, Rafael Gómez, F. Javier de la Mata, Maria Bryszewska

**Affiliations:** 1 Department of General Biophysics, Faculty of Biology and Environmental Protection, University of Lodz, 141/143 Pomorska St., 90-236 Lodz, Poland; katarzynahw@yahoo.es (K.H.); barbara.klajnert@biol.uni.lodz.pl (B.K.-M.); maria.bryszewska@biol.uni.lodz.pl (M.B.); 2 Institute of Biophysics and Cell Engineering of NASB, 27 Akademicheskaya St., 220072 Minsk, Belarus; viktar.abashkin@gmail.com; 3 Leibniz Institute of Polymer Research Dresden, 6 Hohe St., 01069 Dresden, Germany; 4 Department of Organic and Inorganic Chemistry and Research Chemistry Institute “Andrés M. del Río” (IQAR)., University of Alcalá, 28871 Alcalá de Henares, Spain; cornelia.pena@uah.es (C.E.P.-G.); javier.sancheznieves@uah.es (J.S.-N.); rafael.gomez@uah.es (R.G.); javier.delamata@uah.es (F.J.d.l.M.); 5 Networking Research Center for Bioengineering, Biomaterials and Nanomedicine (CIBER-BBN), 28029 Madrid, Spain; 6 Institute “Ramón y Cajal” for Health Research (IRYCIS), 28034 Madrid, Spain

**Keywords:** silver nanoparticles, carbosilane dendrons, siRNA, complexation, anticancer gene therapy

## Abstract

Gene therapy is a promising approach in cancer treatment; however, current methods have a number of limitations mainly due to the difficulty in delivering therapeutic nucleic acids to their sites of action. The application of non-viral carriers based on nanomaterials aims at protecting genetic material from degradation and enabling its effective intracellular transport. We proposed the use of silver nanoparticles (AgNPs) surface-modified with carbosilane dendrons as carriers of anticancer siRNA (siBcl-xl). Using gel electrophoresis, zeta potential and hydrodynamic diameter measurements, as well as transmission electron microscopy, we characterized AgNP:siRNA complexes and demonstrated the stability of nucleic acid in complexes in the presence of RNase. Hemolytic properties of free silver nanoparticles and complexes, their effect on lymphocyte proliferation and cytotoxic activity on HeLa cells were also examined. Confocal microscopy proved the effective cellular uptake of complexes, indicating the possible use of this type of silver nanoparticles as carriers of genetic material in gene therapy.

## 1. Introduction

Despite many years of intensive research, no satisfactory anticancer therapy has been developed so far. Gene therapy is one of the most promising approaches, based on the delivery of therapeutic nucleic acid molecules with different mechanisms of action in cancer cells. One of the proposed methods involves inhibiting or modulating gene expression by introducing small interfering RNAs (siRNAs) that specifically target and neutralize mRNA associated, e.g., with cell proliferation or their response to chemotherapeutic agents [1,2].

One of the biggest challenges of gene therapy involves the efficient delivery of siRNA inside the target cells. This is due to the specificity of nucleic acids, their low stability, susceptibility to enzymatic degradation, and negative charge hindering transport across cell membranes [3]. Therefore, research is directed at developing effective carriers of therapeutic nucleic acids that could overcome these obstacles [4]. Much attention in this regard is devoted to cationic nanoparticles, capable of forming stable non-covalent complexes with negatively charged nucleic acids. The application of such formulations provides protection of genetic material against degradation, as well as its efficient transport in the bloodstream and into the cells, which can significantly enhance the therapeutic effect [5].

Here, we focused on the possibility of using spherical silver nanoparticles (AgNPs) surface-modified with carbosilane dendrons as carriers of anticancer siRNA. The work was primarily aimed at in vitro characterization of the complexes, their stability in the presence of nucleotide enzymes, and their effect on human cells, including cytotoxicity and transfection efficacy.

## 2. Results

### 2.1. Characterization of AgNP:siRNA Complexes

**Figure 1:**
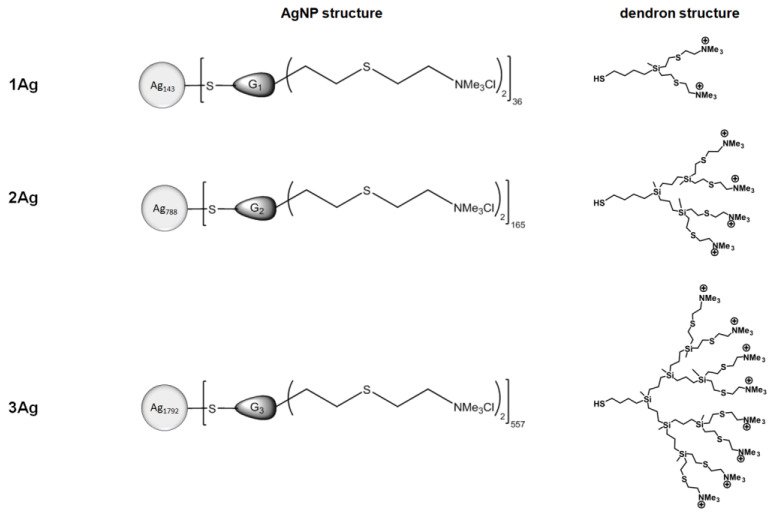
Chemical structures of the studied AgNPs.

**Figure 2:**
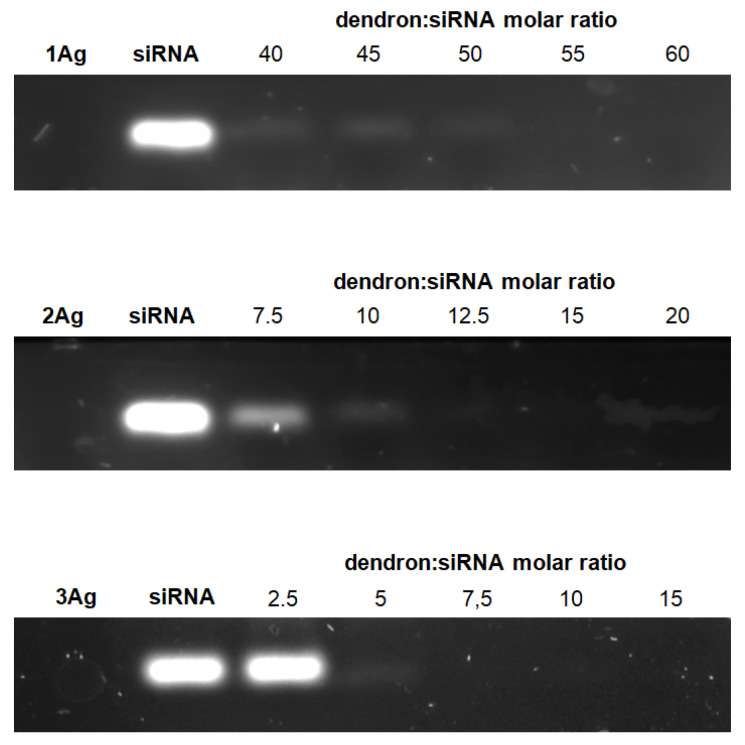
Electrophoresis of AgNP:siRNA complexes at varying dendron:siRNA molar ratios.

The first step of the investigation involved the characterization of complexes of dendronized silver nanoparticles (Figure 1, Table 1) and siRNA (siBcl-xl). The ability of AgNPs to form complexes with siRNA as a function of the dendron:siRNA molar ratio was evaluated by electrophoresis in 3% agarose gel (Figure 2). Free siRNA in the gel is visible due to the GelRed intercalation. Nucleic acid in complex with silver nanoparticles cannot be stained with the dye or migrate in a gel in a degree comparable to free siRNA. Therefore, as the concentration of nanoparticles in the mixture increases, the amount of free nucleic acid decreases and thus the fluorescent signal associated with dye intercalation. This phenomenon enabled the determination of dendron:siRNA molar ratios at which the nucleic acid is completely complexed with silver nanoparticles (55:1 for 1Ag, 12.5:1 for 2Ag, and 7.5:1 for 3Ag). The molar ratio decreased with the increase of the generation of dendrons on the surface of silver nanoparticles.

**Figure 3:**
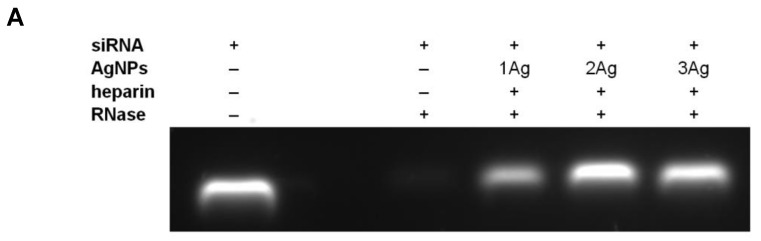

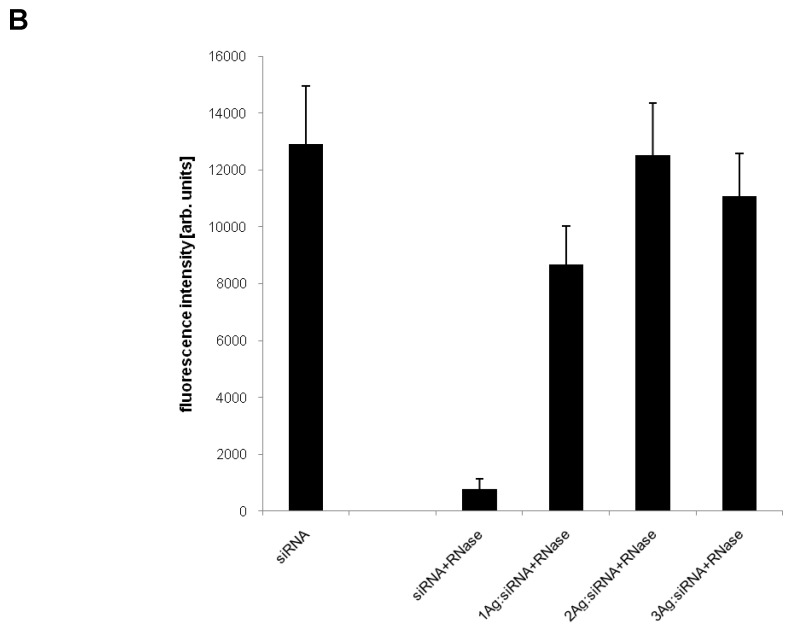
Protective effect of AgNPs towards siRNA (siBcl-xl) treated with RNase. (**A**) The first lane demonstrates migration of non-complexed siRNA. The third lane shows migration of siRNA incubated with RNase A/T1 (10 µg/mL) for 30 min at 37 °C. The following lanes show the migration of siRNA released from complexes with AgNPs, indicating the protection of siRNA from enzymatic cleavage. (**B**) Quantification (by computer image analysis using ImageJ software) of bands in the gel. Data presented as the percentage of band intensity, mean ± SD, *n* = 3.

Further, we showed that the complexation with AgNPs provides protection of siRNA from the action of RNase (Figure 3). Free nucleic acid in the presence of enzyme is almost completely degraded, whereas incubation with silver nanoparticles protects siRNA from RNase (the addition of heparin before electrophoresis led to siRNA release from the complexes and its migration in the gel).

**Figure 4:**
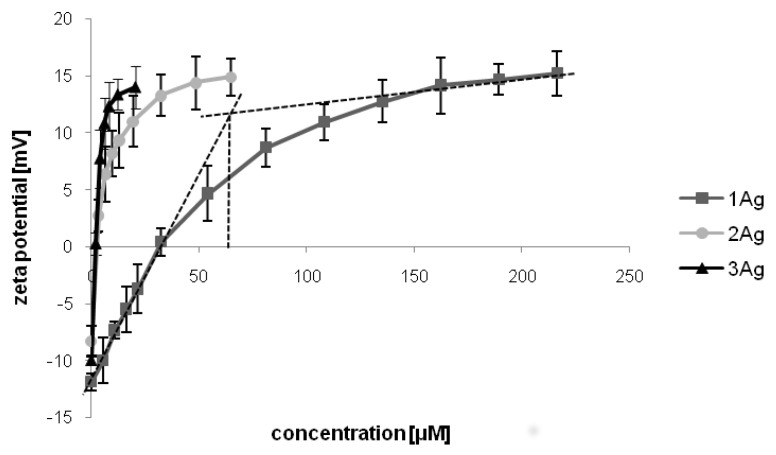
Changes in the zeta potential during the titration of siRNA (siBcl-xl) with AgNPs. The abscissa values refer to the dendron concentration. Data presented as mean ± SD, *n* = 3 (8 measurements each). The dashed lines illustrate the determination of the maximum dendron:siRNA ratio in a complex with the use of the two-tangent method.

To confirm the dendron:siRNA molar ratios determined by gel electrophoresis and to assess the surface electrostatic potential of complexes, we performed titration with zeta potential measurement (Figure 4). Titration curves showed surface potential change due to nucleic acid complexation with the studied AgNPs. Upon the addition of silver nanoparticles to the siRNA solution, the initial zeta potential of free nucleic acid (approximately –12 mV) increased with an increasing concentration of AgNPs, until reaching a plateau at approximately +15 mV. The curves were used to determine the binding stoichiometry (using the two-tangent method). The number of dendrons on the surface of AgNPs per one siRNA molecule equaled ~60 for 1Ag, ~11 for 2Ag, and ~6 for 3Ag, which correlated with the dendron:siRNA molar ratios determined by electrophoresis. Further, these experiments allowed the positive surface electrostatic potential of the studied complexes to be shown.

**Figure 5:**
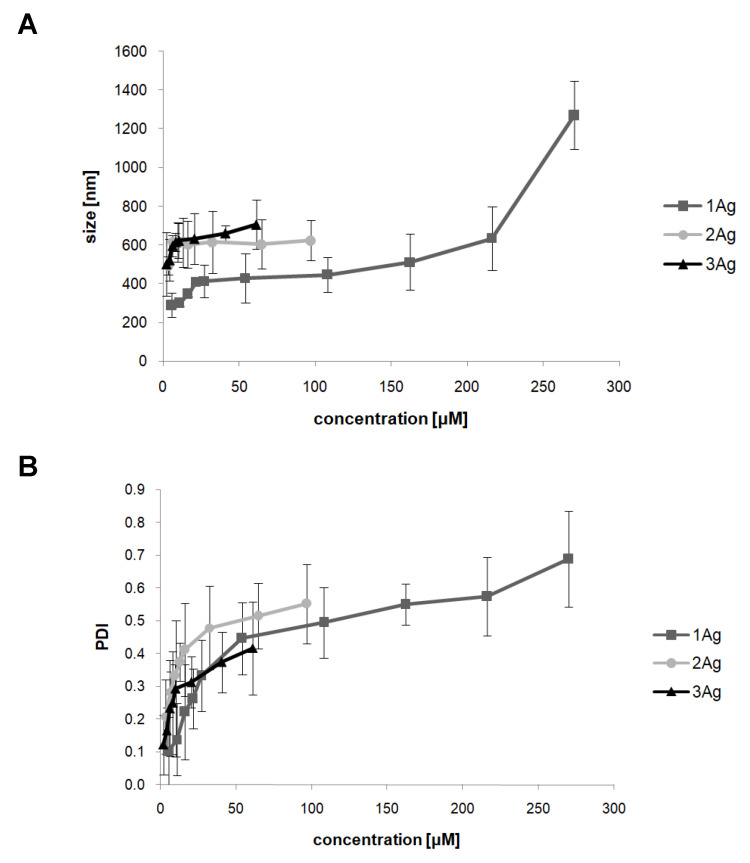
Changes in the hydrodynamic diameter (**A**) and polydispersity index (**B**) of AgNP:siRNA (siBcl-xl) complexes as a function of increasing concentrations of AgNPs. The abscissa values refer to the dendron concentration. Data presented as mean ± SD, *n* = 3 (8 measurements each).

The hydrodynamic diameter of complexes was assessed by the dynamic light scattering (DLS) method (Figure 5). The size of the complexes in the dendron:siRNA molar ratios determined by electrophoresis equaled ~430 nm (PDI ~0.45) for 1Ag, ~614 nm (PDI ~0.37) for 2Ag, and ~615 nm (PDI ~0.25) for 3Ag. Additional experiments for complexes in higher dendron:siRNA molar ratios showed a marked increase in the hydrodynamic diameter of complexes accompanied by higher PDI values, suggesting the formation of aggregates.

**Figure 6:**
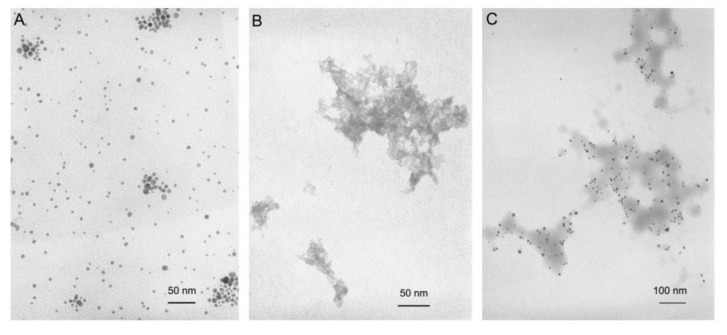
Ultrastructure of 2Ag nanoparticles (**A**), siRNA (siBcl-xl) (**B**), and 2Ag:siRNA (siBcl-xl) complexes prepared in a 12.5:1 dendron:siRNA molar ratio (**C**).

The morphological structure of 2Ag:siRNA complexes prepared in a 12.5:1 dendron:siRNA molar ratio was visualized by transmission electron microscopy (TEM) (Figure 6), which confirmed the size and heterogeneous organization of the complex assessed by hydrodynamic diameter measurements.

### 2.2. Impact of Dendronized Silver Nanoparticles and AgNP:siRNA Complexes on Human Cells

**Figure 7:**
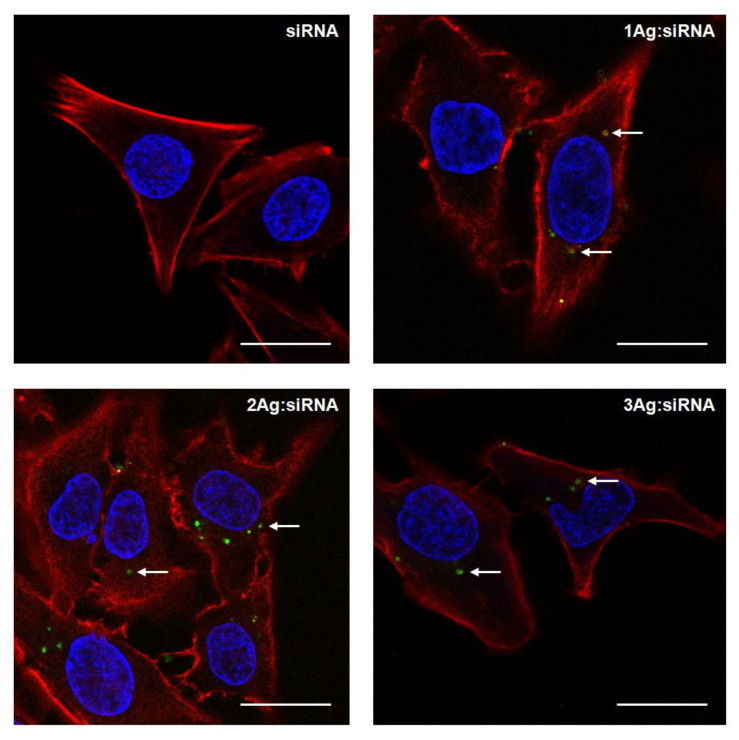
Confocal microscopy images of HeLa cells after 24 h of incubation with free FITC-labeled siRNA (siBcl-xl, 100 nM) or its complexes with 1Ag, 2Ag, and 3Ag prepared in dendron:siRNA molar ratios of 55:1, 12.5:1, and 7.5:1, respectively. Bar = 10 μm. White arrows indicate FITC-labeled siRNA inside the cells.

The ability of AgNPs to deliver siRNA into HeLa cells was proved by confocal microscopy (Figure 7), with FITC-siRNA inside the cells being visible as green spots. This technique also showed that the treatment with complexes at given concentrations does not change the morphology of the cells. Thus, the next step involved the characterization of the hemolytic and cytotoxic activity of the studied nanoparticles and their complexes with siRNA over the range of concentrations.

**Figure 8:**
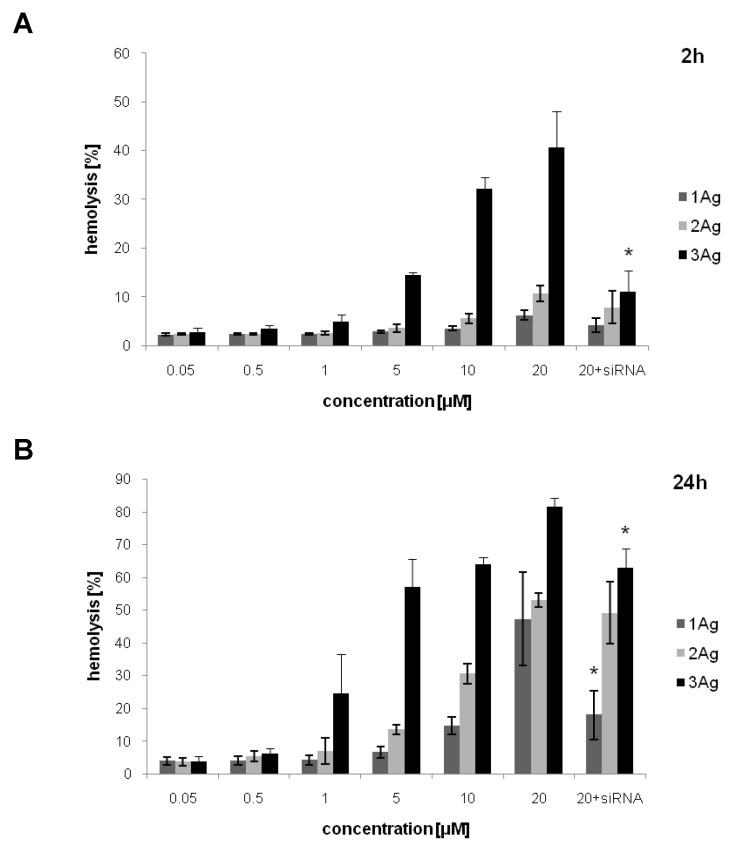
Hemolysis induced by AgNPs and their complexes with siRNA (siBcl-xl) after 2 (**A**) and 24 h of incubation (**B**). The abscissa values refer to the dendron concentration. Data presented as the percentage of hemolysis, mean ± SD, *n* = 4. * Statistically significant difference compared to free nanoparticle at *p* < 0.05.

Hemolysis induced by silver nanoparticles and AgNP:siRNA complexes depended on the concentration of tested compounds and the incubation time (Figure 8). The degree of hemolysis increased with the increase of dendron generation; it was also higher after a longer incubation time. The complexes showed lower hemolytic activity compared to free AgNPs in the case of 1Ag (after 24 h) and 3Ag (after 2 and 24 h).

**Figure 9:**
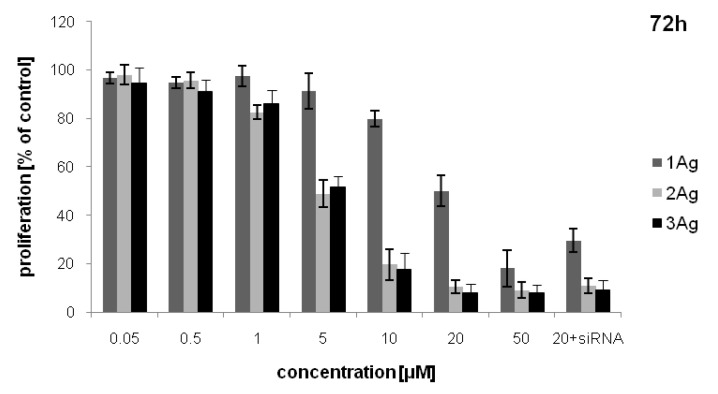
Inhibition of lymphocyte proliferation by AgNPs and their complexes with siRNA (siBcl-xl) after 72 h of treatment. The abscissa values refer to the dendron concentration. Data presented as percentage of viability of control (PHA-treated) cells, mean ± SD, *n* = 3.

Inhibition of lymphocyte proliferation also depended on dendron generation. The weakest inhibiting activity was observed for 1Ag, while for 2Ag and 3Ag the effect was comparable. There was no statistically significant difference between the activity of the AgNP:siRNA complexes and free nanoparticles at the same dendron concentrations (Figure 9).

**Figure 10:**
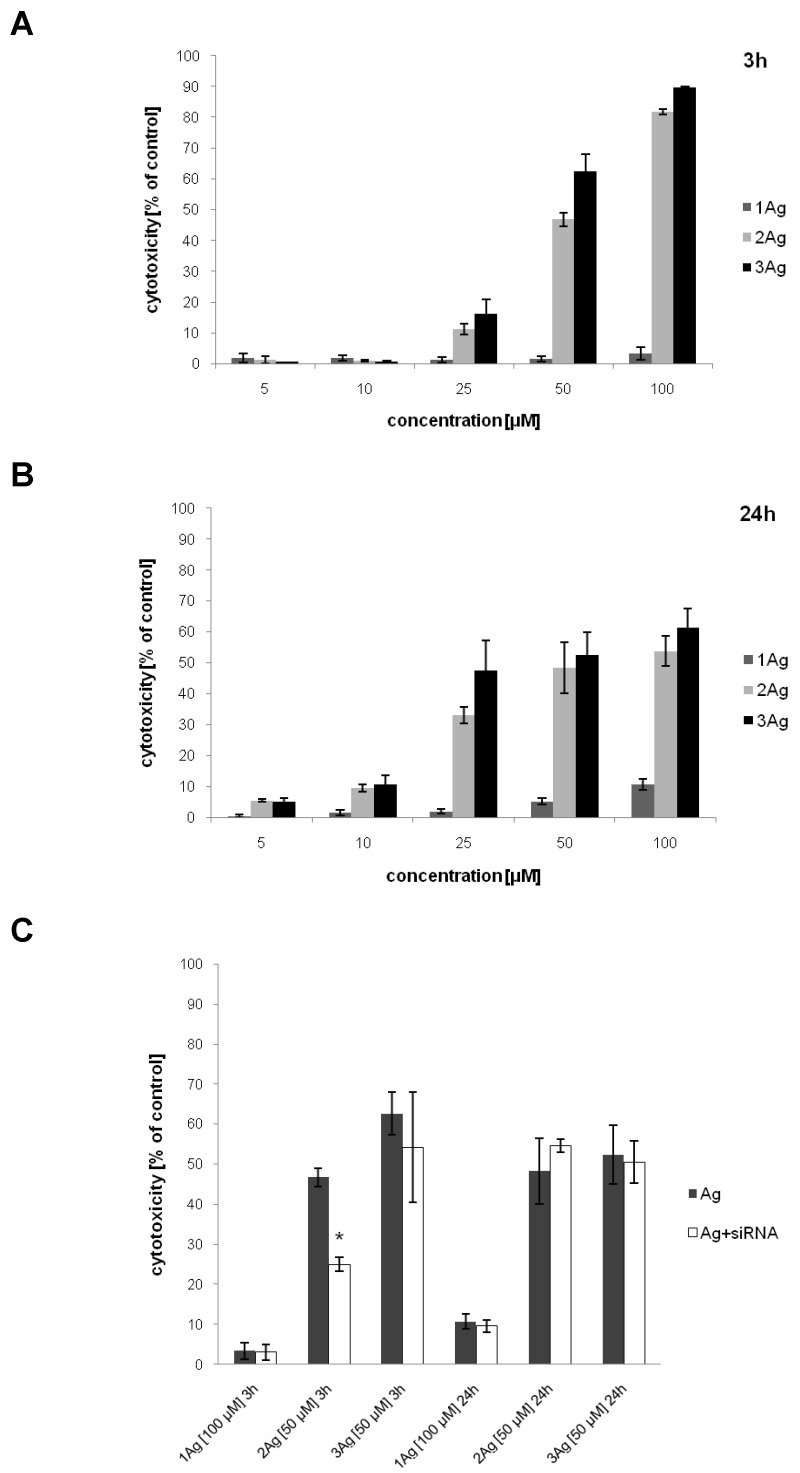
Cytotoxic effect of AgNPs (**A**,**B**) and their complexes with siRNA (siBcl-xl) (**C**) on HeLa cells. Viability was determined by the LDH assay after 3 and 24 h of treatment. The abscissa values refer to the dendron concentration. Data presented as the percentage of cytotoxicity compared to control (untreated) cells, mean ± SD, *n* = 3. * Statistically significant difference compared to free nanoparticle at *p* < 0.05.

The cytotoxicity of the tested compounds was evaluated on HeLa cells (Figure 10). Similarly to the hemolysis studies, the cytotoxic activity depended on the concentration of the tested compounds and incubation time, increasing in the case of 1Ag and decreasing for 2Ag and 3Ag between 2 and 24 h of incubation. The cytotoxic effect increased with the dendron generation (in the case of 1Ag, it was lower compared to 2Ag and 3Ag (Figure 10A,B)). No difference in cytotoxicity was observed comparing the complexes and free nanoparticles at the same dendron concentration; only for 2Ag after 3 h of incubation the complex showed significantly lower activity (Figure 10C).

## 3. Discussion

One of the most promising approaches in modern gene therapy exploits RNA interference (RNAi), a biological process in which RNA molecules inhibit gene expression by targeting and neutralizing mRNA. This effect may be mediated by two types of small RNA molecules, microRNA (miRNA) and siRNA [6]. Since its discovery in 1990s, RNAi quickly attracted the attention of scientists due to the wide range of potential applications in the treatment of viral infections, neurological disorders, and cancer. The use of RNAi in the therapy of the latter is particularly interesting, providing a more specific approach to reduce tumor growth by targeting cancer-related genes, enhance the sensitivity of cancer cells to chemotherapeutics, or inhibit cell invasion and migration [1,2].

In order to exhibit an anticancer effect, therapeutic RNA molecules must be efficiently transported into the cell to reach its molecular targets. This step is a major obstacle for the development of effective gene therapy due to the negative charge, structural stiffness, and high sensitivity to enzymatic degradation of nucleic acids. These features significantly hamper the transport of nucleic acids in the bloodstream, across cellular membranes, and between intracellular compartments [3,7,8]. Thus, effective delivery systems are currently being sought to overcome these barriers. An appropriate carrier for a therapeutic nucleic acid should primarily provide its protection against the activity of nucleases, extended blood half-life, efficient and specific transport to the tumor area and into the target cells, as well as intracellular release [4].

Initially, viruses seemed to be the best candidates for carriers in gene therapy because of their innate ability to introduce nucleic acids into eukaryotic cells, providing efficient transfection and long-term silencing of gene expression. However, it soon became apparent that viral carriers have serious drawbacks due to the high cost of production, along with the induction of immune responses and generation of various side effects, lowering the therapeutic effect. Thus, researchers began to look for non-viral delivery systems [9,10,11].

At present, the most commonly studied non-viral carriers for nucleic acids are based on nanomaterials, including liposomes, carbon nanotubes, or dendrimers. However, their application, despite a high transfection efficacy and facile production, remains limited because of low stability, weak targeting potential, and difficult in vivo tracking [12,13]. Metal nanoparticles constitute an interesting alternative due to their unique size- and shape-dependent physicochemical properties [14,15]. These include high storage stability, as well as significant reactivity and large surface area, enabling a wide range of functionalization of nanoparticles. Appropriate selection of surface modifications may further increase the stability, bioavailability, and targeting potential of metal nanoparticles, but most importantly, provide a positive surface electrostatic potential, enabling interactions with negatively charged nucleic acid molecules. This may enable the formation of stable non-covalent complexes, capable of intracellular delivery of nucleic acids and their protection against enzymatic degradation [16]. Therapeutic nucleic acids can be attached directly to metal nanoparticles by covalent bonds. This solution, however, might significantly affect their structure and hinder release inside the cell [17]. For the purpose of generating a surface positive charge, metal nanoparticles were modified with quaternary ammonium, poly(ethyleneimine) (PEI), triethylenetetramine (TETA), chitosan, amino acids, etc. [16,17,18]. Non-covalent complexes based on electrostatic interactions showed enhanced delivery and increased anticancer activity, with the strength of these effects dependent mainly on the nanoparticle:nucleic acid ratio and surface electrostatic potential of the systems [17].

In the present work, we explored the possibility of using silver nanoparticles surface-modified with carbosilane dendrons as carriers of anticancer siRNA. Although gold nanoparticles (AuNPs) have been more thoroughly tested for the delivery of therapeutic nucleic acids due to high stability and resistance to oxidation, silver nanoparticles can be a valuable alternative due to their lower price and higher reactivity, increasing the range of possibilities of surface functionalization [16,19,20]. Since cationic carbosilane dendrimers of low cytotoxicity were previously shown to form stable complexes with siRNA [21,22], increasing its intracellular delivery and toxic effect [23], we chose carbosilane dendrons as a coating of spherical AgNPs. Such silver nanoparticles (also additionally modified with polyethylene glycol (PEG) chains) were studied only for their antibacterial and antifungal properties [24,25,26]; therefore, this is the first report on their application as carriers of siRNA.

Carbosilane dendrons of different generations and dendron-coated gold nanoparticles were previously characterized in terms of their interactions with plasma proteins [27,28], induction of hemolysis, effect on platelet aggregation, and lymphocyte proliferation [29]. Interestingly, their ability to limit HIV replication in infected PBMCs by intracellular delivery of siRNA Nef was also examined; however, neither dendron:siRNA nor AuNP:siRNA complexes showed enhanced activity compared to free nucleic acid [29].

In this study, we assessed the formation, stoichiometry, and properties of complexes of studied AgNPs and anticancer siRNA (siBcl-xl) (as a function of the dendron concentration), their ability to protect nucleic acid from enzymatic degradation, and to deliver it inside cancer cells. The effect of free nanoparticles and complexes on human cells (erythrocytes, PBMCs, and HeLa) was also evaluated. We chose anti-Bcl-xl siRNA since this anti-apoptotic protein is frequently overexpressed in many tumors and plays a crucial role in cancer progression [30], making it a good target for gene therapy [31,32].

Using gel electrophoresis, the method commonly applied for the analysis of nucleic acids’ complexation by nanoparticles [29,33,34,35], we determined the dendron:siRNA molar ratios corresponding to the maximum saturation of siRNA by AgNPs. As anticipated, the dendron:siRNA molar ratios decreased with increasing nanoparticle/dendron generation, which correlated with the increase in the number of dendron’s positively charged –NMe_3_^+^ groups (Figure 1, Table 1), indicating the formation of complexes based on electrostatic interactions. The complexes also showed the ability to protect siRNA against RNase activity, which was previously observed for different nanoparticles [36,37].

The stoichiometric ratios of complexes determined by gel electrophoresis were confirmed by measurement of changes in the zeta potential during titration of siRNA with AgNPs [37,38]. Further, this assay showed the positive surface electrostatic potential of complexes, which was also demonstrated for dendriplexes [39,40,41], including those formed by carbosilane dendrimers and siBcl-xl [22,42]. This feature is crucial for the intracellular delivery of the cargo, since positively charged nanosystems can penetrate cell membranes more effectively than neutral or negatively charged ones [43]. It could also suggest that siRNA is covered with nanoparticles; however, considering the molar concentration of the whole AgNP instead of dendron in the complex, it is clear that there is roughly one or less silver nanoparticles per one siRNA molecule. This may be partially explained by nucleic acids wrapping around positively charged spherical metal nanoparticles, thereby creating a chromatin-like structure [44].

The hydrodynamic diameters of the complexes, supported by TEM images, are relatively large (~500 nm), and high values of the polydispersity index, especially at higher dendron:siRNA molar ratios, indicate the formation of aggregates [45,46]. It should be noted, however, that the size determined by DLS is usually larger than that observed by TEM due to the increased light scattering by bigger particles, which shifts the measured particle size towards higher values [29]. TEM images are therefore more accurate. Nevertheless, it was previously shown that dendriplexes based on poly(amidoamine) (PAMAM), phosphorus, and carbosilane dendrimers, characterized by similar sizes, can effectively transport nucleic acids into the cells [22,23,40,47].

The therapeutic application of nanoparticles often involves intravenous administration. Therefore, it is crucial to characterize their effects on blood cells before starting any in vivo testing. AgNPs surface-modified with carbosilane dendrons showed concentration- and dendron generation-dependent hemolysis and inhibition of lymphocyte proliferation. In case of hemolysis, the effect was also time-dependent. The nanoparticles showed high hemolytic activity after 2 h of incubation above the 20 μM (for 1Ag and 2Ag) and 5 μM (for 3Ag) dendron concentration, which indicates the possibility of the application of AgNP:siRNA complexes in the lower, non-hemolytic range of the dendron concentration. In addition, siRNA complexation reduced the level of hemolysis (especially in the case of 3Ag). Similar results were obtained for free carbosilane dendrons and dendron-modified AuNPs, with minor discrepancies most likely due to differences in the structure of nanoparticles and concentration/distribution of dendrons on their surface [29]. From these results, we postulate that the observed effects are related to dendrons, their charge, and generation, and to a lesser extent to the silver core of nanoparticles. Although the toxicity of non-modified metal nanoparticles was reported to be size- and shape-dependent, the data are inconsistent. On the other hand, the surface functionalization significantly influences their properties, including cytotoxicity, cellular uptake, and biodistribution [17,48]. Although a positive surface charge of nanoparticles improves the efficacy of gene transfer and drug delivery, a higher cytotoxicity of such systems was reported due to the disruption of cellular membranes’ integrity, damage of mitochondria, generation of reactive oxygen species (ROS), etc. [49]. The AgNP:siRNA complexes also inhibited lymphocyte proliferation and caused hemolysis, although for the latter the effect was less visible compared to free nanoparticles. This is most probably also associated with the positive charge on the surface of the complexes. Interestingly, the incubation of carbosilane dendrons and dendronized AuNPs with human serum albumin (HSA) limited their hemolytic effect [29]. This supports the hypothesis regarding the role of positively charged dendrons in the induction of hemolysis, and indicates potentially lower impact of carbosilane dendron-coated nanoparticles on blood cells in vivo due to their interactions with plasma proteins.

The cytotoxic activity of dendronized AgNPs on HeLa cells was also dendron generation-dependent, with 1Ag being the least toxic. The cytotoxicity of 2Ag and 3Ag was lower after 24 h of incubation compared to that observed after 3 h, which may be related to the specificity of the LDH assay and cell growth. In this case, the LDH leakage from damaged cells was measured, whereas intact cells (including untreated control) continued to divide, resulting in underestimated values after prolonged incubation. This observation further confirms the mechanism of cytotoxicity based primarily on membrane damage by positively charged nanoparticles. The use of other cytotoxicity test, such as MTT, neutral red, or resazurin assay, could show the toxicity increasing over time.

Despite intracellular delivery of siRNA by AgNPs (demonstrated by confocal microscopy), no increase in cytotoxic activity of AgNP:siRNA complexes was found compared to free nanoparticles. This may be due to several factors, including hampered endosomal escape after endocytosis, limited release of siRNA from complexes, or an inadequate amount of intracellularly delivered siRNA to exert its cytotoxic effect [42]. On the other hand, the expression of Bcl-xl and other proteins of Bcl-2 family in HeLa cells is relatively low ([50,51], www.proteinatlas.org), which at the present stage makes it impossible to draw unequivocal conclusions about the effectiveness of the proposed siRNA delivery vehicles. This in turn encourages further work both on the delivery system and the selection of appropriate therapeutic nucleic acids for specific cellular models.

## 4. Materials and Methods

### 4.1. Dendronized Silver Nanoparticles

AgNPs surface-modified with cationic carbosilane dendrons of different generations (Figure 1, Table 1) were synthesized and characterized as described previously [24].

### 4.2. Short Interfering RNA

Non-ﬂuorescent and fluorescein (FITC)-labeled anti-Bcl-xl siRNAs (siBcl-xl) (sense: 5′CAGGGACAGCAUAUCAGAGdTdT3′, antisense: 5′CUCUGAUAUGCUGUCCCUGdTdT3′) were synthesized (Dharmacon Inc., Lafayette, CO, USA) and dissolved in RNase-free water prior to experimental use.

### 4.3. Characterization of AgNP:siRNA Complexes

#### 4.3.1. Gel Electrophoresis

Complexes of siRNA (1.5 μM) and AgNPs in different dendron:siRNA molar ratios were prepared in 10 mM phosphate buffer, pH 7.4, and incubated for 20 min at room temperature. Then the samples were placed on 3% agarose gel containing GelRed stain (Biotium, Fremont, CA, USA) and separated by electrophoresis in Tris-acetate-EDTA (TAE) buffer for 45 min at 90 V/35 mA. The gel was subsequently visualized using UV light and a digital picture of the stained gel was taken with UVP ChemiDoc-It2™ Imager (Thermo Fisher Scientific, Waltham, MA, USA).

To study the protection of siRNA against enzymatic degradation as a result of complexation with AgNPs, the complexes in dendron:siRNA molar ratios of 55:1, 12.5:1, and 7.5:1 were prepared for 1Ag, 2Ag, and 3Ag, respectively. The samples were incubated with RNase A/T1 (Thermo Fisher Scientific, Waltham, MA, USA, 10 μg/mL) for 30 min at 37 °C and 10 min on ice. Heparin (Sigma Aldrich, St. Louis, MO, USA, 0.082 mg/mL) was added to the samples for 10 min to release siRNA from the complexes. Samples prepared in this way were analyzed by electrophoresis in a manner analogous to that described above.

Band intensities were quantified digitally using ImageJ software and presented as mean ± SD, *n* = 3.

#### 4.3.2. Size and Zeta Potential Measurements

Measurements of size and zeta potential were performed with the use of a Zetasizer Nano ZS (Malvern Instruments Ltd., Malvern, UK). Complexes were prepared in phosphate-buffered saline (PBS) by mixing siRNA (1 μM) with an increasing concentration of nanoparticles (different dendron:siRNA molar ratios), placed in the low-volume sizing cuvettes (ZEN0112, Malvern Instruments Ltd., Malvern, UK) for size determination or in the folded capillary cells (DTS 1070, Malvern Instruments Ltd., Malvern, UK) for zeta potential measurements at 37 °C. The data were analyzed using Malvern software. Particle size distribution was determined by a multimodal peak analysis. When the polydispersity index (PDI) was lower than 0.5, Z-average was taken into account; when PDI was higher than 0.5, individual peaks were analyzed. Data were presented as mean ± SD, *n* = 3 (8 measurements each).

#### 4.3.3. Transmission Electron Microscopy

A complex of siRNA (3 µM) and 2Ag (in 12.5:1 dendron:siRNA molar ratio) was prepared in 10 mM phosphate buffer, pH 7.4, and 15 µL of sample placed on 200 mesh copper grid with a carbon-coated surface for 10 min. The sample was stained with 2% uranyl acetate for 20 min, then washed with deionized water and dried at room temperature. Images were taken with a JEOL JEM-1010 transmission electron microscope (JEOL Ltd., Tokyo, Japan) at 80 kV.

### 4.4. Cell Culture

The HeLa (cervix adenocarcinoma) human cell line was purchased from ATCC (Manassas, VA, USA) and maintained under standard conditions in DMEM Medium (Thermo Fisher Scientific, Waltham, MA, USA) supplemented with 10% fetal bovine serum, penicillin (100 U/mL), and streptomycin (100 µg/mL) (Sigma-Aldrich, St. Louis, MO, USA) at 37 °C in an atmosphere of 5% CO_2_. Cells were sub-cultured 2-3 times per week.

Blood from healthy donors from the Central Blood Bank (Lodz, Poland) was anticoagulated with 3% sodium citrate. Erythrocytes were separated from blood plasma and leukocytes by centrifugation (4000× *g*, 10 min, 4 °C), washed three times with PBS, and used immediately after isolation.

PBMCs (peripheral blood mononuclear cells) were isolated from blood samples using Histopaque 1077 (Sigma Aldrich, St. Louis, MO, USA) gradient (1500× *g*, 15 min, 24 °C) and cultured in RPMI-1640 Medium (Thermo Fisher Scientific, Waltham, MA, USA) supplemented with 10% fetal bovine serum, penicillin (100 U/mL), and streptomycin (100 µg/mL) (Sigma-Aldrich, St. Louis, MO, USA) at 37 °C in an atmosphere of 5% CO_2_.

### 4.5. Cellular Uptake Studies: Confocal Microscopy 

HeLa cells were seeded into 6-well plates at a density of 1 × 10^5^ cells per well and treated with free siRNA-FITC (100 nM) and its complexes with 1Ag, 2Ag, and 3Ag prepared in PBS in dendron:siRNA molar ratios of 55:1, 12.5:1, and 7.5:1, respectively, for 24 h. The cells were subsequently washed with PBS, fixed with 4% formaldehyde for 30 min, and stained with fluorescent dyes (DAPI (Sigma Aldrich, St. Louis, MO, USA) for 5 min for nuclei visualization and Texas Red™-X Phalloidin (Thermo Fisher Scientific, Waltham, MA, USA) for 20 min for actin visualization). Images were obtained with a Leica TCS SP8 microscope (Leica Microsystems GmbH, Wetzlar, Germany) at different excitation wavelengths (DAPI: 405 nm, FITC: 495 nm, Phalloidin: 565 nm). Leica software was used to analyze the data.

### 4.6. Hemotoxicity

AgNPs in a dendron concentration range of 0.05–20 μM, and AgNP:siRNA complexes prepared in PBS in dendron:siRNA molar ratios of 55:1, 12.5:1, and 7.5:1 for 1Ag, 2Ag, and 3Ag, respectively, were added to the erythrocytes (at 2% hematocrit). The concentration of AgNPs in complexes was constant and equaled 20 μM. The samples were incubated at 37 °C for 2 and 24 h, centrifuged (3000× *g*, 10 min, 4 °C), and their absorbance was measured at 540 nm using a PowerWave HT Microplate Spectrophotometer (BioTek Instruments Inc., Winooski, VT, USA). The percentage of hemolysis was calculated using the following formula: H(%) = (A 540 nm/A water 540 nm) × 100%.

Data were presented as percentage of hemolysis, mean ± SD, *n* = 4.

### 4.7. Lymphocyte Proliferation

PBMCs were seeded into 96-well black plates at a density of 1 × 10^5^ cells per well. To induce the proliferation of lymphocytes, PBMC were stimulated with phytohemagglutinin (PHA, Sigma Aldrich, St. Louis, MO, USA, 10 μg/mL).

The cells were treated with AgNPs in a dendron concentration range of 0.05–50 μM, and AgNP:siRNA complexes prepared in PBS in dendron:siRNA molar ratios of 55:1, 12.5:1, and 7.5:1 for 1Ag, 2Ag, and 3Ag, respectively, for 72 h. The concentration of AgNPs in complexes was constant and equaled 20 μM. Following the incubation, resazurin was added to the culture medium to a final concentration of 10 µg/mL and the plates were incubated at 37 °C in darkness to allow conversion of resazurin to resorufin. Fluorescence of metabolized resazurin was measured after 30 and 90 min at 530-nm excitation and 590-nm emission using a PowerWave HT Microplate Spectrophotometer (BioTek Instruments Inc., Winooski, VT, USA). Cell viability was calculated as the increase in resorufin fluorescence between 30 and 90 min. Lymphocyte proliferation was presented as the percentage of viability of control (PHA-treated) cells, mean ± SD, *n* = 3.

### 4.8. Cytotoxicity Assay

HeLa cells were seeded into 96-well transparent plates at a density of 1 × 10^4^ cells per well. The cells were treated with AgNPs in a dendron concentration range of 5–100 μM, and AgNP:siRNA complexes prepared in PBS in dendron:siRNA molar ratios of 55:1, 12.5:1, and 7.5:1 for 1Ag, 2Ag, and 3Ag, respectively, for 3 and 24 h. The concentration of AgNPs in complexes was constant and equaled 100 (1Ag) or 50 μM (2Ag and 3 Ag).

Following the incubation, the LDH assay was performed using a Pierce™ LDH Cytotoxicity Assay Kit (Thermo Fisher Scientific, Waltham, MA, USA) according to the manufacturer’s protocol. The absorbance at 490 and 680 nm was measured using a PowerWave HT Microplate Spectrophotometer (BioTek Instruments Inc., Winooski, VT, USA). To determine the LDH release, the 680-nm absorbance value was subtracted from the 490-nm absorbance value. The percentage of cytotoxicity was calculated using the following formula: cytotoxicity(%) = (compound-treated LDH release—spontaneous LDH release)/(maximum LDH release—spontaneous LDH release)

Data were presented as the percentage of viability of control (untreated) cells, mean ± SD, *n* = 3.

### 4.9. Statistics

Statistical analysis was performed using Statistica (StatSoft Inc., Tulsa, OK, USA). The normality of distribution was assessed using the Shapiro–Wilk test and homogeneity of variance using the Brown–Forsythe test. Analysis of significance was performed using Kruskal–Wallis test with Dunn’s post hoc test. In all tests, *p* values < 0.05 were considered statistically significant.

## 5. Conclusions

In this work, we proved that silver nanoparticles surface modified with carbosilane dendrons form stable complexes with siRNA, protecting it from enzymatic degradation and ensuring efficient cellular uptake. However, both the nanoparticles and complexes showed hemolytic and cytotoxic activity, which excludes their intravenous administration at the higher concentrations tested. The complexes did not show enhanced anticancer activity; therefore, further studies are needed involving different therapeutic siRNA or other cell lines showing varied expression of Bcl-2 family proteins. The use of other dendron:siRNA ratios could also be considered, giving complexes with a lower positive surface charge, thereby reducing the detrimental effects on cells.

## Figures and Tables

**Table 1:** Characteristics of the studied AgNPs.

AgNP	Molecular Formula	Molecular Weight (g/mol)
**1Ag**	Ag_143_(C_19_H_45_Cl_2_N_2_S_3_Si)_36_	33,309.85
**2Ag**	Ag_788_(C_41_H_97_Cl_4_N_4_S_5_Si_3_)_165_	255,383.86
**3Ag**	Ag_1792_(C_85_H_201_Cl_8_N_8_S_9_Si_7_)_557_	1,365,420.42
